# Biological soil crust development affects bacterial communities in the *Caragana microphylla* community in alpine sandy areas

**DOI:** 10.3389/fmicb.2023.1106739

**Published:** 2023-03-15

**Authors:** Hong Zhou, Lun Li, Yunxiang Liu

**Affiliations:** ^1^State Key Laboratory of Plateau Ecology and Agriculture, Qinghai University, Xining, China; ^2^Qinghai Academy of Agricultural and Forestry Sciences, Xining, China; ^3^Qilian Mountain National Park Qinghai Service Guarantee Center, Xining, China

**Keywords:** biological soil crusts, developmental process, bacterial community, Illumina sequencing, Gonghe basin

## Abstract

**Introduction:**

Biological soil crusts (BSCs) constitute a substantial portion of primary production in dryland ecosystems. They successionally mature to deliver a series of ecosystem services. Bacteria, as an important community in BSCs, play critical roles in maintaining the structure and functions of BSCs. However, the process by which bacterial diversity and community are altered with BSC development is not fully understood.

**Methods:**

In this study, amplicons sequencing was used to investigate bacterial diversity and community compositions across five developmental stages of BSCs (bare sand, microbial crusts, algae crusts, lichen crusts, and moss crusts) and their relationship with environmental variables in the Gonghe basin sandy land in Qinghai-Tibet Plateau, northwestern China.

**Results:**

The results showed that Proteobacteria, Actinobacteria, Cyanobacteria, Acidobacteria, Bacteroidetes, and Firmicutes were predominant in different developmental stages of BSCs, accounting for more than 77% of the total relative abundance. The phyla of Acidobacteria and Bacteroidetes were abundant in this region. With BSC development, bacterial diversity significantly increased, and the taxonomic community composition significantly altered. The relative abundance of copiotrophic bacteria, such as Actinobacteria, Acidobacteria, Bacteroidetes, Verrucomicrobia, Planctomycetes, and Gemmatimonadetes significantly increased, whereas the relative abundance of oligotrophic bacteria, such as Proteobacteria and Firmicutes significantly decreased. The relative abundance of Cyanobacteria in the algae crusts was significantly higher than that in the other developmental stages (*p* < 0.05).

**Conclusion:**

Variations in bacterial composition suggested that the potential ecological functions of the bacterial community were altered with BSC development. The functions varied from enhancing soil surface stability by promoting soil particle cementation in the early stages to promoting material circulation of the ecosystem by fixing carbon and nitrogen and decomposing litter in the later stages of BSC development. Bacterial community is a sensitive index of water and nutrient alterations during BSC development. SWC, pH value, TC, TOC, TN, NO_3_^−^, TP and soil texture were the primary environmental variables that promoted changes in the bacterial community composition of BSCs.

## 1. Introduction

Drylands are extremely important for achieving global sustainability as they cover more than 40% of the land surface on Earth and host more than a third of the total human population (Reynolds et al., 2007). Approximately half of arid and semiarid drylands are devoid of plants and are instead occupied by biological soil crusts (BSCs), an assemblage of soil fine particles, cyanobacteria, green algae, lichen, mosses and microbes in various proportions (Belnap, 2001). BSCs support a wide range of ecosystem functions, such as enhancing soil stability, reducing soil and wind erosion, improving the nutrient cycling of ecosystems, regulating water availability and redistribution, and influencing the emergence and survival of vascular plants (Belnap and Lange, 2003; Maestre et al., 2011; Wang et al., 2021; Barrera et al., 2022). Given the global distribution of BSCs and their key functional roles in the ecosystems where they are prevalent, understanding the developmental mechanisms and ecological functions of BSCs is critical in formulating sustainable natural resource management and conservation policies in drylands.

Biological soil crusts colonize bare grounds and thus constitute the first successional stage in the development of arid and semiarid area ecosystems (Garcia-Pichel et al., 2003). BSCs are categorized into main successional stages according to the predominant organisms present, including microbial, algae, lichen and moss crusts (Zhang et al., 2015; Liu et al., 2017). Microbial and algae crusts represent the earliest developmental stages of biocrusts, whereas lichen and mosses appear during the later stages. Bacteria play an important role in the formation and development of BSCs (Belnap, 2001). In arid and semiarid areas, many drought-tolerant and high temperature-resistant bacteria (up to 70°C) can colonize the sand surface, alter the physical and chemical properties of the soil through their physiological metabolic mechanisms, and lay a strong foundation for the formation and development of BSCs (Jorge-Villar and Edwards, 2013). Additionally, some bacterial phyla, such as Bacteroides and Cyanobacteria, can secrete metabolites or form centimeter-long filament bundles to maintain the physical structure of BSCs (Garcia-Pichel and Wojciechowski, 2009). Moreover, as primary producers, bacteria (primarily cyanobacteria) increase soil fertility by fixing carbon and nitrogen in the atmosphere, providing a strong foundation for the development of BSCs (Wang et al., 2021).

Bacterial communities present in BSCs have been investigated in many studies worldwide. Among them, owing to the significant colonization status, cyanobacteria in BSCs were studied most frequently (Steven et al., 2014; Zhang et al., 2018). However, the trends in the variation of bacterial diversity with BSC development remain inconclusive. Evidence from some studies suggested that bacterial diversity gradually increased with the development of BSCs (Gundlapally and Garcia-Pichel, 2006; Zhang et al., 2016), whereas that from other studies suggested that bacterial diversity peaked in the middle stages of BSC development and gradually declined thereafter (Redfield et al., 2006; Liu et al., 2017). In addition, significant changes occurred in the composition of the microbial community with the development of BSCs. Specifically, Cyanobacteria was the most abundant phylum in the algae and lichen crusts (Nagy et al., 2005; Abed et al., 2010; Zhang et al., 2016), whereas the abundances of Proteobacteria and Bacteroidetes were higher in the moss crusts (Maier et al., 2014; Zhang et al., 2016). Nevertheless, changes in the bacterial community composition with the development of BSCs are not fully understood. Findings from previous studies also highlighted that the bacterial community in BSCs is primarily affected by cyanobacterial abundance, soil water content, soil particle composition, and electrical conductivity (Zhang et al., 2018; Zhou et al., 2020). However, the results of the study widely varied based on the study area. In recent years, owing to the development of high-throughput sequencing technology, the accuracy of microbial component surveys has considerably improved (Atrache, 2017; Li et al., 2020). This approach facilitates a more precise analysis of the shift in microbial populations at different developmental stages of BSCs, and also makes the analyses of key ecological driving factors feasible.

Alpine sandy land regions, such as the Qinghai-Tibet Plateau, are recognized as regions very sensitive to climate change (Wang et al., 2010). Gonghe Basin, located in the northeastern part of the Qinghai-Tibet Plateau, forms an important ecological barrier in northwest China. *Caragana microphylla* is one of the most typical sandy shrubs found in the sandy land of Gonghe Basin and plays an important role in desertification control and biodiversity conservation (Cheng et al., 2022). BSCs are widely distributed in the *C. microphylla* community. Until now, only a few studies conducted in these regions have discussed the BSC carbon flux, its response to climate change, and the effects of BSCs on moisture absorption in soil, among other parameters (Jia et al., 2016; Cheng et al., 2019). However, our understanding of bacterial community varies with BSC development in the regions remains limited. Studies of this knowledge gap could improve our understanding of BSC developmental mechanism and functions in the process of ecological restoration of sandy land, and are critical to predicting alpine desert ecosystem responses under climate change scenarios.

In this study, 16S rRNA gene amplicon sequencing was used to systematically explore the bacterial diversity and community composition at the different developmental stages of BSCs in the Gonghe basin sandy land in northwestern China. We aimed to answer the following questions: (i) What is the uniqueness of the BSC bacterial communities in this study area (alpine sandy land)? (ii) Do microbial taxa change with the development of BSCs, and how? and, (iii) What are the key environmental variables affecting bacterial communities of BSCs?

## 2. Materials and methods

### 2.1. Study site

**Figure 1:**
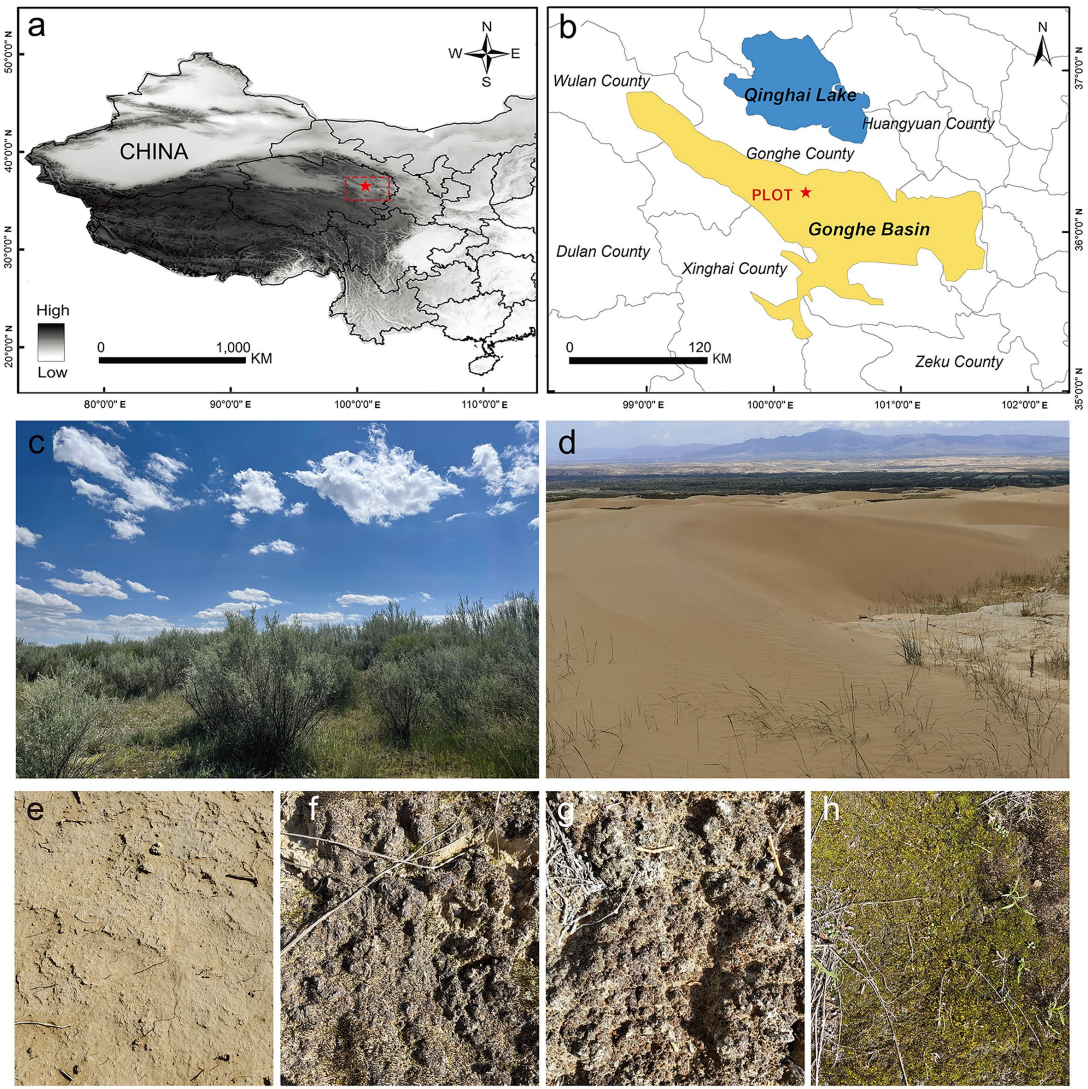
Study site. **(A,B)** Geographical location of the sampling plot in the Gonghe Basin, western China; **(C)** revegetation using desert shrubs for stabilizing mobile dunes; **(D–H)** bare sand, microbial crusts, algae crusts, lichen crusts, and moss crusts, respectively.

The study site (100°25′ E, 36°24′ N) is located in the northwest of the Gonghe Basin sandy land, in Gonghe County, Qinghai Province. It is managed by the Qinghai Republican Desert Ecosystem Research Station, National Forestry and Grassland Administration (Figure 1). The Gonghe Basin (~13,800 km^2^) is the area of the Qinghai-Tibet Plateau that is most severely affected and threatened by land desertification; 33.5% of the basin area has undergone desertification. The average altitude of the Gonghe Basin is more than 2,900 m. This area is characterized by a plateau mountain climate, with a mean annual temperature of 1.0–5.2°C and mean annual precipitation of 311.1–402.1 mm. At our study site, the annual mean temperature is 2.4°C and the annual average precipitation is 246.3 mm, with approximately 70% of precipitation occurring between May and September. The mean annual potential evaporation is 1,716.7 mm. Soil type is aeolian sand and soil salinity is 28.7 g/kg. The Gonghe Basin sandy land comprises fixed, semi-fixed, and shifting dunes, with vegetation cover rates of 30–50, 10–30, and <10%, respectively. The vegetation type is temperate desert shrubland. Shrubs primarily consist of cultivated vegetation, and *Caragana korshinskii*, *Hippophae rhamnoides*, and *Tamarix chinensis* are the predominant species, with *Lycium chinense* being spatially scattered. Additionally, different types of BSCs, including microbial, algae, lichen, and moss crusts, are widely distributed on the fixed and semi-fixed dunes (with a cover of 40–80%; Figure 1).

### 2.2. Sampling and measurements

Sampling was conducted in August 2021. The experimental region had not received any rainfall in 72 h before sampling. Intact samples included a series of different developmental and successional BSCs. A relatively flat area (approximately 150 m × 200 m) was selected to ensure greater homogeneity in site conditions and reduce the variability associated with small-scale differences. Each developmental stage was considered from three individual plots (at least 20 m between two adjacent plots). Within each subplot, five subsamples were randomly collected and combined to form one composite sample for each BSC developmental stage. Samples were collected from the interspaces between shrubs (0.5 m away from the shrubs) using a sterile cutting ring (diameter of 9.0 cm) according to a protocol reported by Zhou et al. (2020). Bare sand samples were collected from shifting dunes with a thickness of 2 cm. Fifteen types of different developmental BSCs, including bare sand, were collected (five developmental stages × three individual subplots), and the samples were preserved in an ice box. These samples were sieved (2.0 mm mesh) to remove visible roots and stones and categorized in three parts for further analysis. The first part was air-dried, the second part was stored at 4°C to analyze soil properties, and the third part was freeze-dried at −80°C for DNA extraction.

Soil pH was determined using a potentiometer with a pH electrode. A 1:5 soil/water (w/w) suspension was prepared previously. The soil water content (SWC) was determined by oven-drying the soil to a constant mass at 105°C. The total carbon (TC) and total nitrogen (TN) contents were measured by dry combustion using an elemental analyzer (2400II CHN elemental analyzer; Perkin-Elmer, Waltham, MA, United States). Total phosphorus (TP) was digested in 1 mol L^−1^ H_2_SO_4_ after ignition at 550°C in a muffle furnace, and then measured using the molybdate colorimetric method at 880 nm on a spectrophotometer (UV-2550; Shimadzu, Japan; Pang, 2003). Total organic carbon (TOC) was determined using a wet combustion method with a mixture of potassium dichromate (K_2_Cr_2_O_7_) and sulfuric acid (H_2_SO_4_) under external heating (Lemonnier et al., 2010). Nitrate (NO_3_^−^) and ammonium (NH_4_^+^) were extracted using 50 ml of 2 M KCl. After shaking for 30 min, the solution was filtered and analyzed using a continuous flow analyzer (Skalar, Breda, Netherlands). A standard soil hydrometer method (Klute, 1986) was used to determine the sand, silt, and clay composition in the crust samples. Microbial biomass carbon (MBC) and nitrogen (MBN) content in soil were measured using the chloroform fumigation direct extraction method, as described by Witt et al. (2000).

### 2.3. DNA extraction, amplification, and sequencing

DNA extraction was performed using the PowerSoil®DNA Isolation Kit (MO BIO, Carlsbad, CA, United States) per the manufacturer’s instructions. Total DNA was then eluted and dissolved in 100 μl of elution buffer. DNA samples were purified using a 0.8% (w/v) agarose gel. DNA bands were excised from the gel, and DNA was extracted using a gel extraction kit (Bioer, China) and quantified with a NanoDrop™8,000 spectrophotometer (Thermo Scientific, Waltham, MA, United States). Bacterial 16S rRNA gene fragments were PCR-amplified using conserved domain-specific primers 515F (5′-GTGCCAGCMGCCGCGGTAA-3′) and 806R (5′-GGACTA CHVGGGTWTCTAAT-3′), as described by Caporaso et al. (2012). PCR amplification and tag-encoded high-throughput sequencing of 16S genes were conducted at Novogene Company in Beijing, China, using the Illumina HiSeq platform (PE 2500).

Sequence processing, clustering, taxonomic assignments, and biodiversity calculations were performed using the QIIME pipeline.^1^ Both forward and reverse primers were trimmed. Sequences with high quality (length > 200 bp, without ambiguous base “N,” and average base quality score > 30) were used for downstream analyses. Operational taxonomic units (OTUs) were clustered with 97% similarity cut-off using UPARSE (Edgar, 2013), and chimeric sequences were identified and removed using UCHIME (Edgar et al., 2011). Taxonomic assignments were performed using the SILVA database as a reference (Pruesse et al., 2007). The non-bacterial sequences were discarded. Lastly, to correct the bias caused by different sequencing depths, the sequence data were normalized to 13,509 sequences per sample.

### 2.4. Statistical analysis

Bacterial alpha-diversity was assessed based on the observed species (species richness), the Shannon-Wiener and Chao1 indexes. Differences in bacterial community composition (Bray–Curtis dissimilarity) in the different developmental stages of BSCs were assessed using permutational multivariate ANOVA (PerMANOVA) and visualized using principal coordinate analysis (PCoA). After performing normality and equality of variance tests, repeated-measures analysis of variance (ANOVA) was conducted to compare the effects of soil physicochemical characteristics and microbial community composition in the different developmental stages of BSCs. Post-hoc analyses were performed using Fisher’s least significant difference (LSD) test. Differences were considered to be statistically significant at *p* < 0.05. Mantel tests based on the Bray–Curtis distances were performed to correlate the microbial communities with environmental variables.

Statistical analyses and figure preparation were performed using R 3.6.0 (R.Team, 2011). We used the ggplot2 package (Wickham, 2011) for generating figures.

## 3. Results

### 3.1. Physicochemical characterization of BSCs

The soil water content did not significantly differ between bare sand and microbial crusts but significantly increased from 2.04% (bare sand) to 3.40% (algae crusts), 8.48% (lichen crusts), and 13.65% (moss crusts) with the development of BSCs (Table 1). Similarly, the TOC, TC, TN, TP, and ammonium (NH_4_^+^) concentrations in the samples also increased in a stepwise manner with BSC development. From bare sand to moss crusts, the TOC, TC, TN, and TP increased 4.61, 4.76, 2.65, and 0.39 times, respectively. In contrast, the concentration of nitrate (NO_3_^−^) and the pH value in moss crusts decreased from that in bare sand (*p* < 0.05). The soil texture clearly differed between bare sand and BSCs. The sand content was the highest in the bare sand sample (95.36%) and decreased in microbial crusts (88.97%), algae crusts (86.64%), lichen crusts (75.54%), and moss crusts (75.33%). On the contrary, the silt and clay contents significantly increased with the development of BSCs (*p* < 0.05).

**Table 1:** Physicochemical characteristics in different developmental stages of BSCs.

	Bare sand	Microbial crusts	Algae crusts	Lichen crusts	Moss crusts
SWC (%)	2.04 ± 0.20**d**	2.00 ± 0.80**d**	3.40 ± 1.58**c**	8.48 ± 1.64**b**	13.65 ± 2.7**a**
pH	7.53 ± 0.01**a**	7.48 ± 0.01**a**	7.45 ± 0.02**a**	7.37 ± 0.03**b**	7.25 ± 0.06**b**
TOC (g·kg^−1^)	1.32 ± 0.30**c**	3.07 ± 0.85**b**	3.94 ± 0.66**b**	7.04 ± 2.35**a**	7.40 ± 0.92**a**
TC (g·kg^−1^)	1.83 ± 0.07**c**	7.57 ± 1.48**b**	8.98 ± 0.74**b**	7.96 ± 0.85**b**	10.54 ± 0.4**a**
TN (g·kg^−1^)	0.20 ± 0.07**c**	0.28 ± 0.02**c**	0.55 ± 0.07**b**	0.53 ± 0.02**b**	0.73 ± 0.03**a**
NO_3_^−^ (10^−4^ g·kg^−1^)	2.54 ± 1.09**a**	1.37 ± 0.45**b**	0.97 ± 0.57**bc**	1.06 ± 0.71**c**	0.55 ± 0.17**c**
NH_4_^−^(10^−4^ g·kg^−1^)	0.40 ± 0.08**c**	0.36 ± 0.03**c**	0.65 ± 0.29**b**	0.50 ± 0.10**b**	1.02 ± 0.09**a**
TP (g·kg^−1^)	0.36 ± 0.02**c**	0.42 ± 0.01**b**	0.44 ± 0.01**b**	0.44 ± 0.02**b**	0.50 ± 0.02**a**
Sand (%)	95.36 ± 0.22**a**	88.97 ± 0.81**b**	86.64 ± 0.77**b**	75.54 ± 4.64**c**	75.33 ± 2.90**c**
Silt (%)	4.12 ± 0.25**d**	9.49 ± 0.08**c**	11.72 ± 0.66**b**	22.02 ± 4.18**a**	22.46 ± 2.65**c**
Clay (%)	0.52 ± 0.07**c**	1.55 ± 0.10**b**	1.64 ± 0.12**b**	2.44 ± 0.47**a**	2.21 ± 0.31**a**

Lowercase boldfaced letters depict significant differences across different developmental stages (*P* < 0.05). SWC, soil water content; TOC, total organic carbon; TC, total carbon; TN, total nitrogen; TP, total phosphorus; NO_3_^−^, nitrate nitrogen; NH_4_^−^, ammonium nitrogen.

### 3.2. Microbial biomass and diversity

Microbial biomass carbon content was lower in the bare sand (36.98 mg/kg) and microbial crusts (34.94 mg/kg), and significantly increased (141.80 mg/kg) in the lichen crusts, and then decreased in the moss crusts (120.67 mg/kg). MBN increased with the development of BSCs and peaked in the moss crusts (from 4.04 mg/kg in bare sand to 21.03 mg/kg in moss crusts), being 5.21 times higher than that in the bare sand (Supplementary Figure S1).

**Figure 2:**
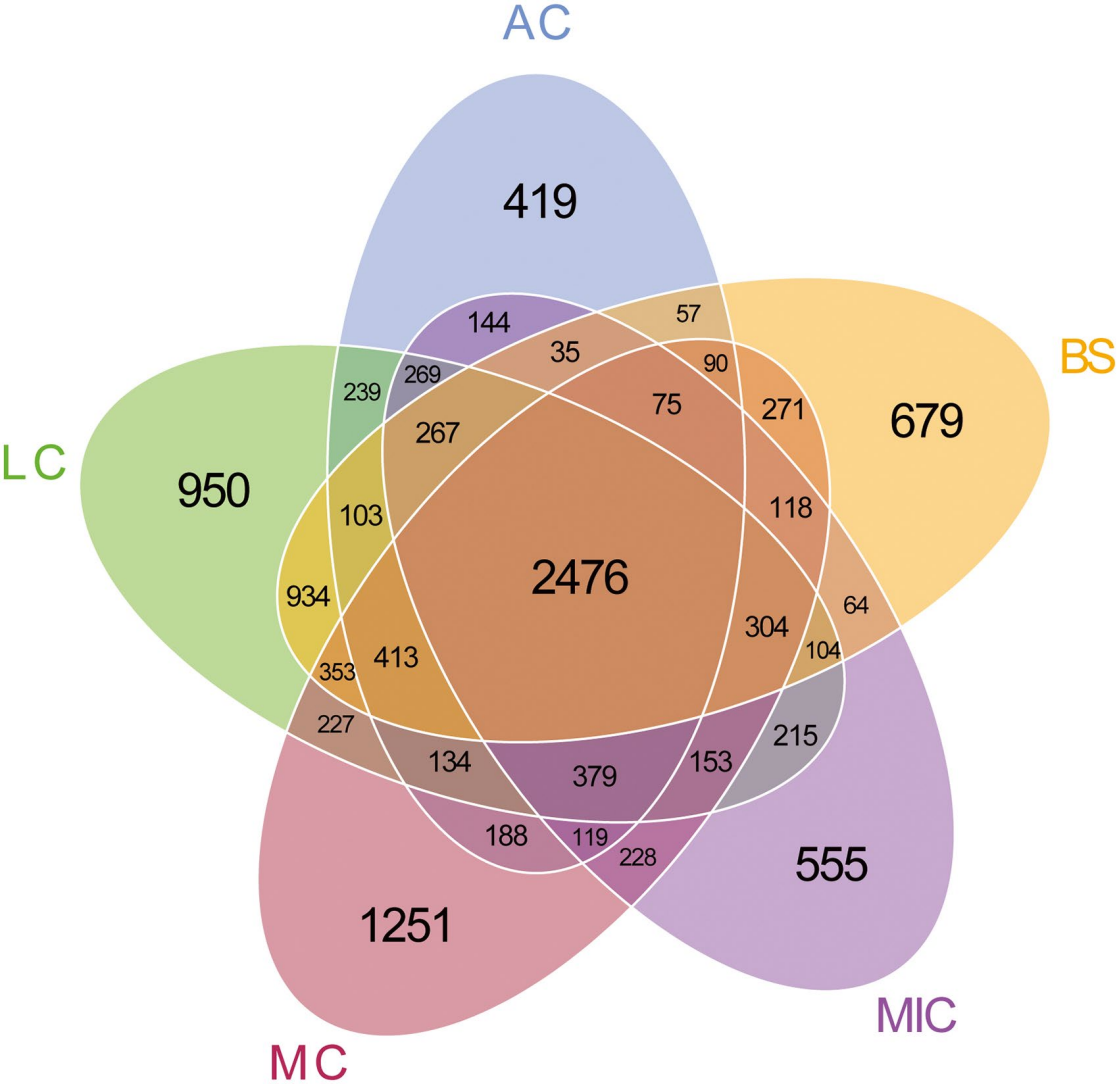
Number of operational taxonomic units (OTUs) of bacteria in each developmental stage of BSCs. BS, bare sand; MIC, microbial crusts; AC, algae crusts; LC, lichen crusts; MC, moss crusts.

**Figure 3:**
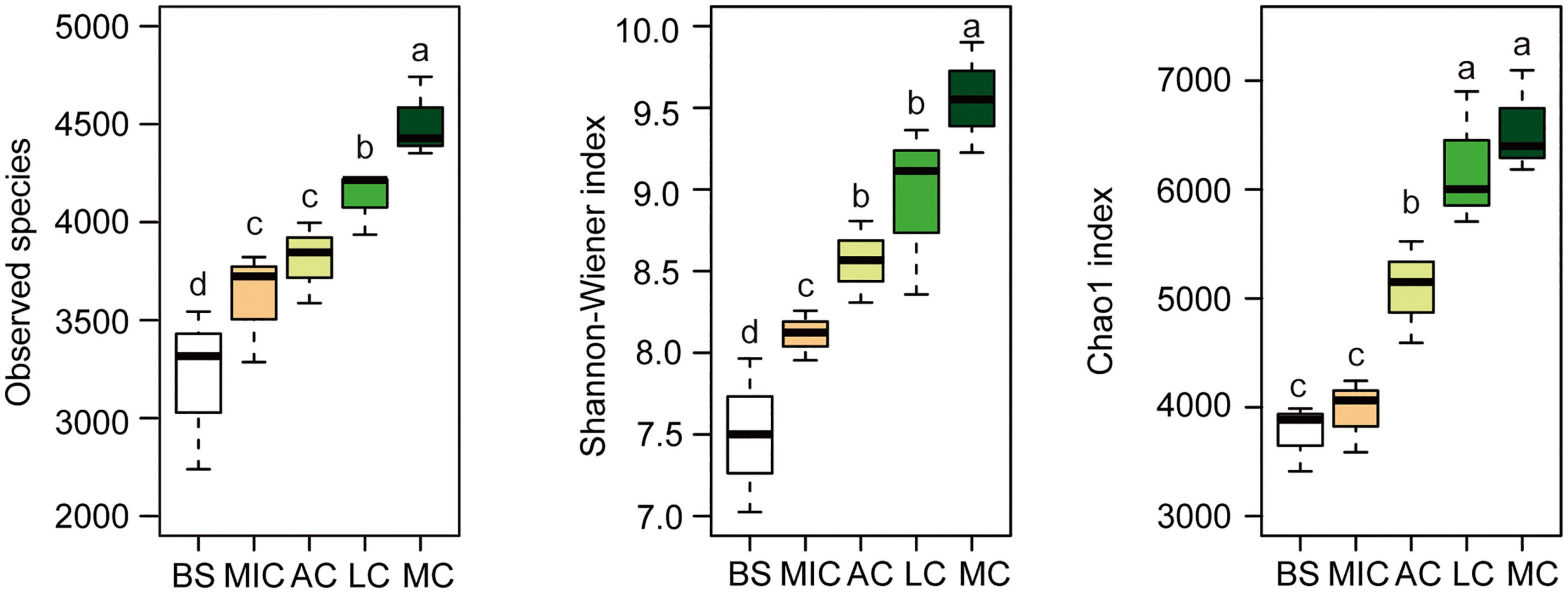
Alpha diversity of the bacterial taxonomic community. The box was drawn to represent values from the lower 1/4 quantile to the upper 1/4 quantile. Whiskers above and below the box represent the upper 95% CI and lower 95% CI, respectively. Medians are indicated using black horizontal bars within boxes. We used one-way ANOVA to observe significant changes between treatments. Letters depict significant differences across compartments. BS, bare sand; MIC, microbial crusts; AC, algae crusts; LC, lichen crusts; MC, moss crusts.

To identify the OTUs that contributed to the divergence in the bacterial community composition in the five developmental stages, we observed overlaps and distinctions in the abundant OTUs in the five developmental stages (Figure 2). As shown in Figure 2, 13,590 bacterial OTUs were detected in all developmental stages of BSCs. There were 2,476 bacterial OTUs common with those in the bare sand and in the different developmental stages of BSCs. Moss crusts were the most abundant in unique OTUs (1,251), followed by lichen crusts (950), bare sand (679), and microbial crusts (555). Algae crusts had the lowest number of unique OTUs (419). Bacterial diversity in different developmental stages of BSCs showed significant differences (Figure 3). The number of observed species significantly increased from 3,200 in bare sand to 3,611 in microbial crusts, 3,810 in algae crusts, 4,124 in lichen crusts, and 4,507 in moss crusts (*p* < 0.05), with increment rates of 12.8, 19.1, 28.9, and 40.8%, respectively. Shannon–Wiener and Chao1 indexes also gradually increased with the development, with the highest values observed in the moss crusts (Figure 3).

### 3.3. Microbial taxonomic composition

**Figure 4:**
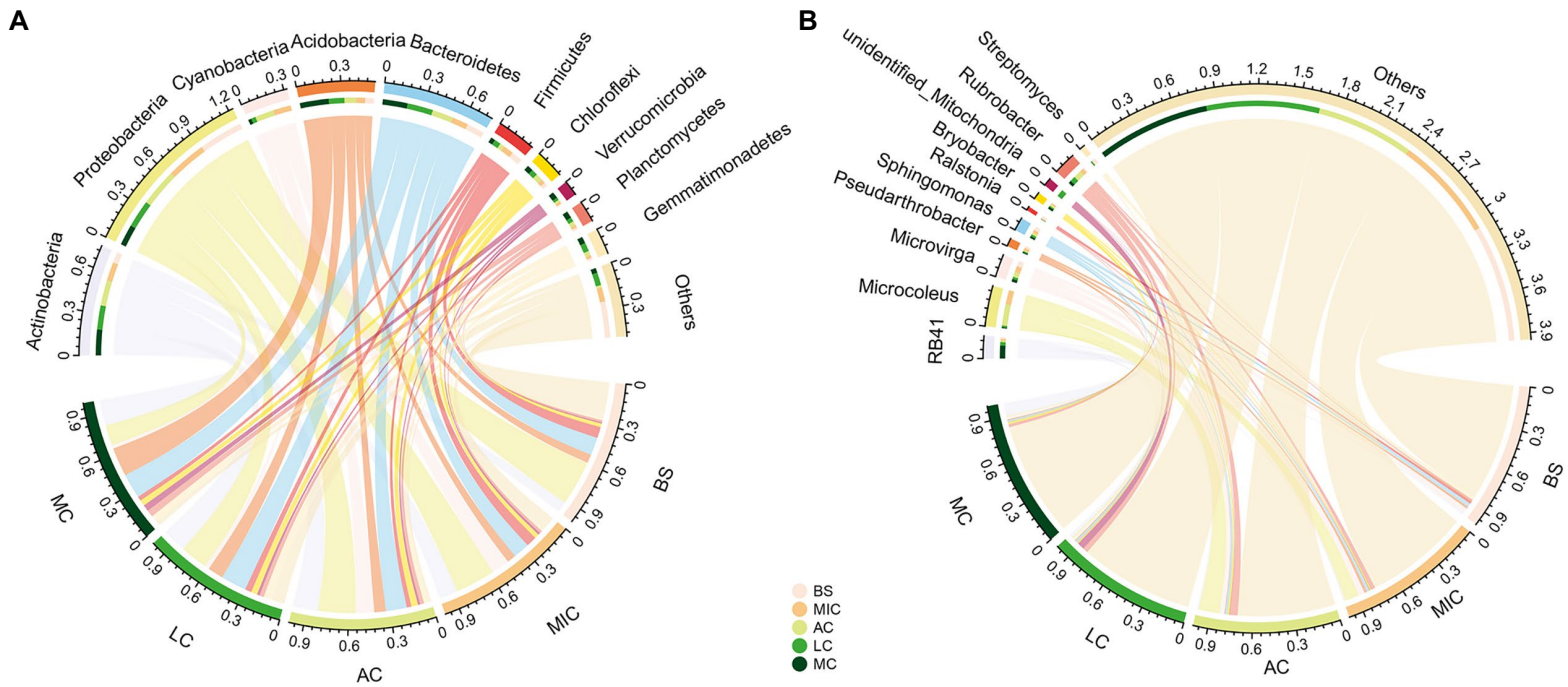
Relative abundance of bacterial communities at the phylum **(A)** and genus **(B)** levels in different developmental stages of BSCs. BS, bare sand; MIC, microbial crusts; AC, algae crusts; LC, lichen crusts; MC, moss crusts.

In total, 58 bacteria phyla were detected in our study. Bacterial communities in all five developmental stages were dominated by Actinobacteria (15.1% ± 4.6%), Proteobacteria (24.6% ± 7.0%), Cyanobacteria (6.5% ± 6.2%), Acidobacteria (10.6% ± 6.0%), Bacteroidetes (15.2% ± 3.0%), and Firmicutes (5.3% ± 2.3%; Figure 4A). The relative abundances of these six phyla accounted for more than 77% of the total relative abundance.

The compositions of bacterial communities distinctly differed in the five developmental stages (Figure 3). Specifically, the relative abundance of Actinobacteria, Acidobacteria, Bacteroidetes, Verrucomicrobia, Planctomycetes, and Gemmatimonadetes significantly increased with the development of BSCs (*p* < 0.05; Figure 4A; Supplementary Table S2). On the contrary, the relative abundance of Proteobacteria and Firmicutes significantly decreased with the development of BSCs. From bare sand to moss crusts, the relative abundance of Proteobacteria decreased from 32.1 to 15.0% and that of Firmicutes decreased from 8.0 to 3.2%. The relative abundance of Cyanobacteria was the lowest in bare sand (0.4% ± 0.1%), significantly increased and peaked (13.3% ± 2.0%) in the algae crusts, and significantly decreased in lichen crusts (3.2% ± 1.0%) and moss crusts (2.3% ± 0.4%). The relative abundance of Chloroflexi showed no significant differences with the development of BSCs (*p* > 0.05; Figure 4A; Supplementary Table S2).

To further investigate taxonomic compositions of different BSC bacterial communities, we compared the dominant bacterial taxa in the different developmental stages of BSCs at the finer levels of classification. At the genus level, 825 genera were identified. We also observed notable differences among the four developmental stages (Figure 4B; Supplementary Table S2). The abundance of two genera significantly increased with the development of BSCs (*p* < 0.05; Supplementary Table S2), including the acidobacterial genera *RB41* and *Bryobacter*. On the contrary, the abundance of two proteobacterial genera *Ralstonia* and *Sphingomonas* significantly decreased with BSC development (*p* < 0.05). Algae crusts had a significantly greater proportion of the cyanobacterial genera *Microcoleus* than other stages (*p* < 0.05).

### 3.4. Relationships between bacterial communities and the physicochemical characterization of BSCs

**Figure 5:**
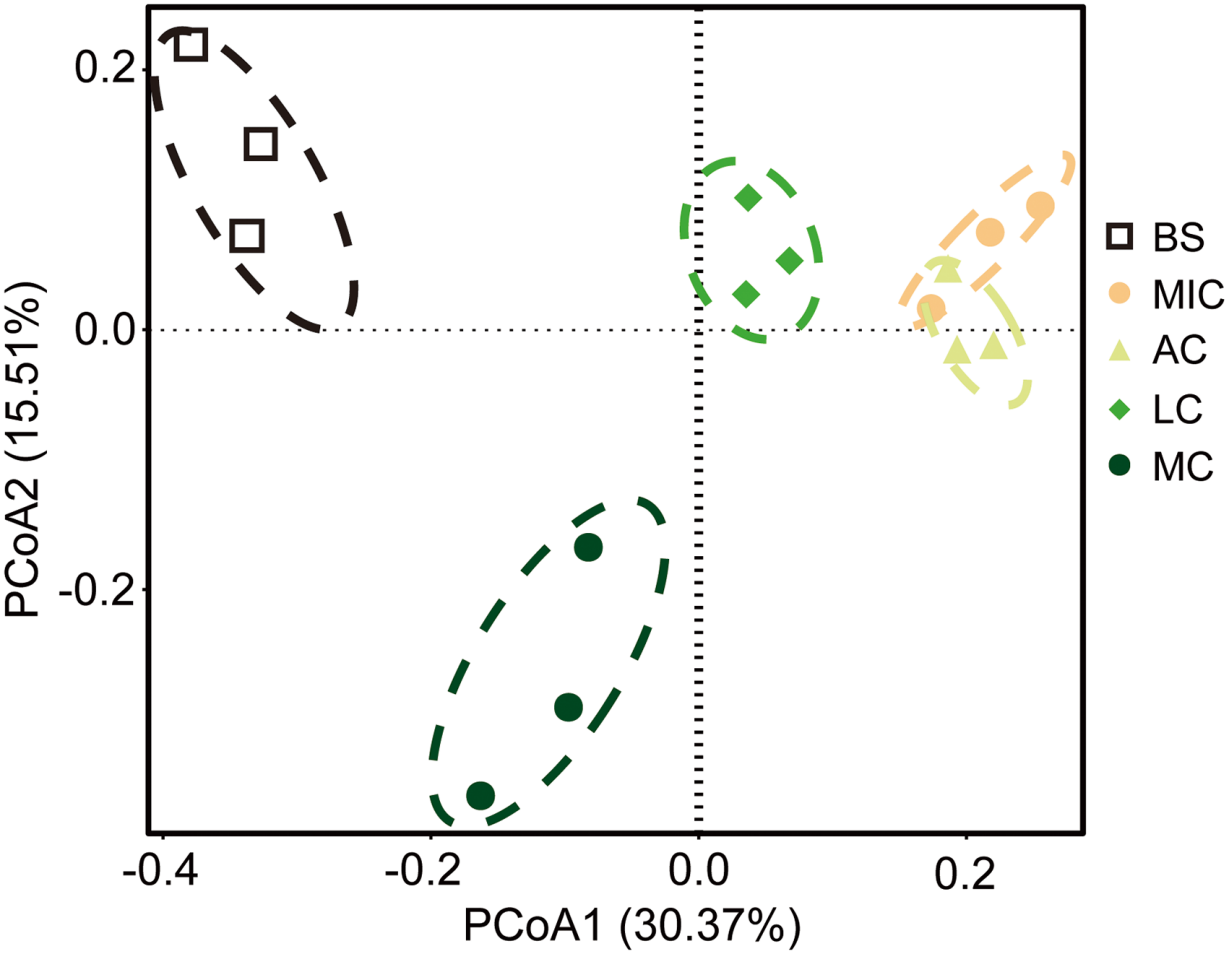
Principal coordinate analysis (PCoA) plots of bacterial communities based on the Bray–Curtis dissimilarities. Circles represent samples from each developmental stage of BSCs. BS, bare sand; MIC, microbial crusts; AC, algae crusts; LC, lichen crusts; MC, moss crusts.

Unconstrained principal coordinate analyses (PCoAs) based on the Bray–Curtis distance matrix of OTU relative abundances were performed to investigate the patterns of bacterial communities (Figure 5). PCoA plots showed that bacterial communities among bare sand and the four developmental stages of BSCs were well separated from each other. Bacterial axes 1 and 2 accounted for 30.37 and 15.51% of the variance, respectively (Figure 5). Results of permutational multivariate analysis of variance (PerMANOVA), Adonis function, confirmed that the bacterial taxonomic communities were significantly different among bare sand and different developmental stages of BSCs (*R*^2^ = 0.422, *p* = 0.001, Supplementary Table S1).

Mantel tests were applied to evaluate the correlations between each environmental factor and bacterial communities in BSCs (Table 2). Among the environmental variables examined, the SWC (*p* = 0.002), pH (*p* = 0.006), TC (*p* = 0.001), TOC (*p* = 0.049), TN (*p* = 0.001), NO_3_^−^ (*p* = 0.008), TP (*p* = 0.001), sand content (*p* = 0.012), silt content (*p* = 0.044), and clay content (*p* = 0.003) were significantly correlated with the bacterial community composition of BSCs. Among these environmental variables, SWC exerted the strongest impact on bacterial community composition (*R* = 0.683). However, the NH_4_^−^ content (*p* = 0.243) showed no significant correlation with the bacterial community composition.

**Table 2:** Mantel tests for determining the bacterial community composition and environmental variables.

Environmental variables	*R*	*P*
SWC (%)	0.683	0.002
pH	0.389	0.006
TOC (g·kg^−1^)	0.206	0.049
TC (g·kg^−1^)	0.598	0.001
TN (g·kg^−1^)	0.388	0.001
NO_3_^−^ (10^−4^ g·kg^−1^)	0.153	0.008
NH_4_^−^(10^−4^ g·kg^−1^)	0.0741	0.243
TP (g·kg^−1^)	0.591	0.001
Sand (%)	0.278	0.012
Silt (%)	0.106	0.044
Clay (%)	0.424	0.003

SWC, soil water content; TOC, total organic carbon; TC, total carbon; TN, total nitrogen; TP, total phosphorus; NO_3_-, nitrate nitrogen; NH_4_-, ammonium nitrogen.

**Figure 6:**
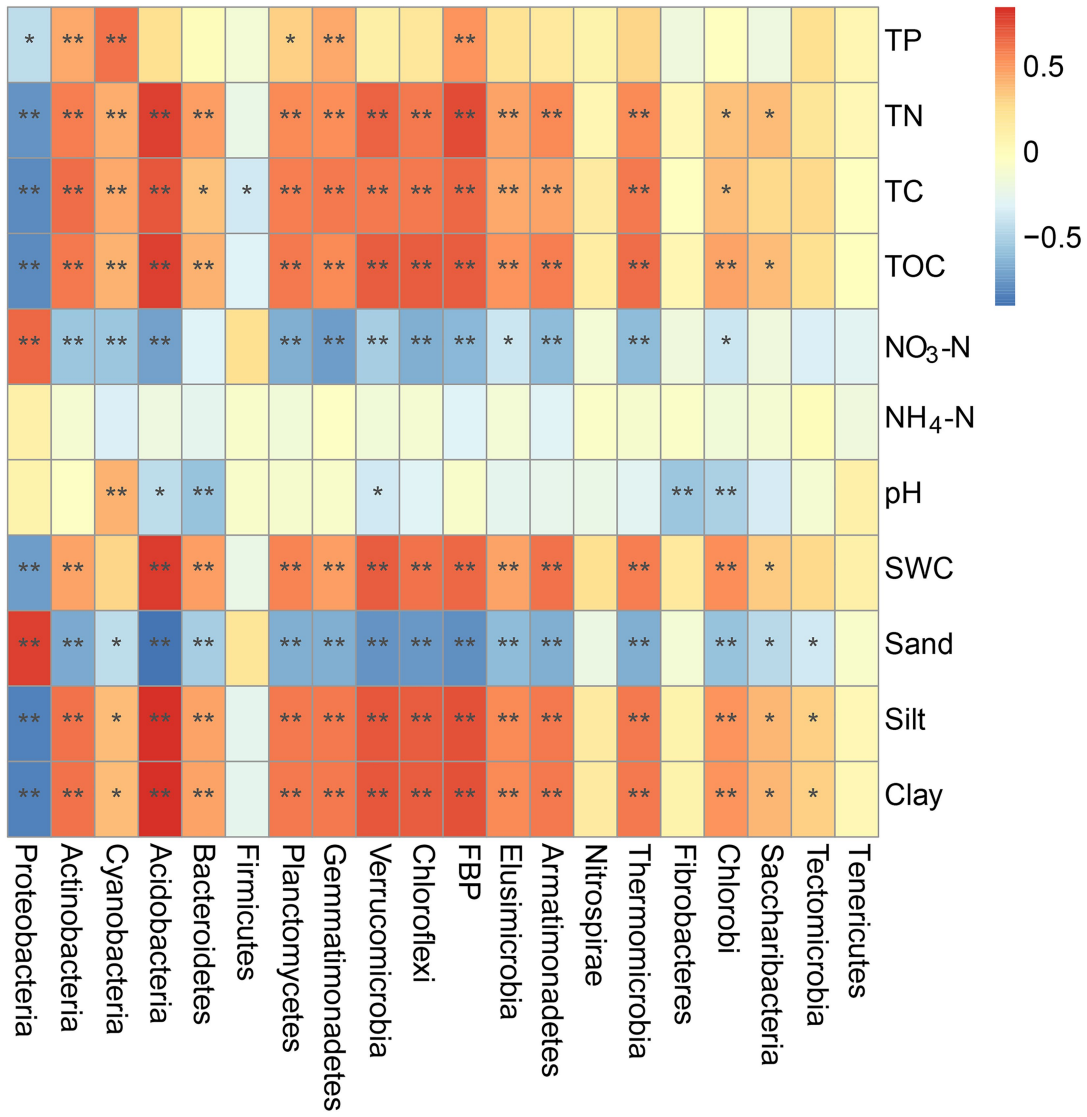
Mantel tests of environmental variables and different bacterial phyla in BSCs. *p < 0.05, **p < 0.01. SWC, soil water content; TOC, total organic carbon; TC, total carbon; TN, total nitrogen; TP, total phosphorus; NO_3_-N, nitrate nitrogen; NH_4_-N, ammonium nitrogen.

Mantel tests were also performed to examine the potential correlations between individual bacterial phyla and environmental variables (Figure 6). For example, the TC, TN, TOC, and clay contents were positively correlated with the compositions of Actinobacteria, Cyanobacteria, Acidobacteria, Bacteroidetes, Planctomycetes, and Gemmatimonadetes but negatively correlated with that of Proteobacteria (*p* < 0.05). Sand and NO_3_^−^ contents in the BSCs were positively correlated with the composition of Proteobacteria (*p* = 0.004 and *p* = 0.017) and negatively correlated with that of other phyla, except Firmicutes (*p* < 0.05). Additionally, the SWC showed significant correlations with the bacterial phyla composition, except Cyanobacteria (Figure 6). TP showed significant positive correlations with the compositions of Actinobacteria, Cyanobacteria, Planctomycetes, and Gemmatimonadetes (*p* < 0.05) but a significant negative correlation with that of Proteobacteria (*p* = 0.013).

## 4. Discussion

### 4.1. Biological soil crusts bacterial communities in alpine sandy land

Given the high phylogenetic diversity, abundance, ubiquity, and biogeochemical importance of microbes (Harris, 2009), studies on microbial communities help us understand how microbes play essential roles in biogeochemical cycling and ecosystem functioning of BSCs. Our data collected from the alpine sandy land of the Qinghai-Tibet Plateau revealed that BSC bacterial communities were diverse and abundant in this region. Higher bacterial diversity in BSCs has been reported in many regions of desert and plateau ecosystems. For example, in the Shapotou area of Tengger Desert (China; average altitude is 1,377 m), the number of OTUs in four different developmental stages of BSCs reached about 4,000, and the Shannon-Wiener indexes were more than 7.2 (Zhang et al., 2018). Here, the total amount of OTUs in BSCs we observed in alpine sandy land was up to 3,600, and their Shannon-Wiener indexes were more than 8.0 (Figure 3), thus much higher than that in the Shapotou area. Ren et al. (2018) observed that soil bacterial alpha diversity was significantly higher at medium altitude (2,060 ~ 3,300 m) than that in low altitudes (less than 2,000 m), which was consistent with our results.

Proteobacteria, Actinobacteria, Cyanobacteria, Acidobacteria, Bacteroidetes, and Firmicutes were predominant in the bacterial communities in BSCs (Figure 4A), similar to observations in the Tengger Desert and Gurbantunggut Desert (Zhang et al., 2016, 2018). However, the relative abundances of Acidobacteria and Bacteroidetes (6.3–20.8% and 11.8–18.6%) in this study were significantly higher than that in Tengger Desert (2.0–9.1% and 2.5–7.9%) and Gurbantunggut Desert (2.0–8.3% and 1.7–5.8%). According to findings from previous studies, the relative abundance of Acidobacteria was significantly unimodal with altitude, with a declining pattern in the middle and low altitudes (1,000 ~ 2,000 m) and an increasing pattern at high altitudes (above 2,000 m; Liu et al., 2016). The altitude of Gonghe Basin (over 2,900 m) was significantly higher than that of the Tengger Desert (1,377 m) and Gurbantunggut Desert (550 m). This could explain the relatively high abundance of Acidobacteria in the BSCs in our study. The phylum Bacteroidetes had a strong ability to adapt to cold environments (Zhang et al., 2009). In addition, Junge et al. (2004) observed that with a decrease in temperature, the relative abundance of Bacteroides increased, which was consistent with our results. An increase in altitude usually corresponds to a change in the hydrothermal conditions, with a higher altitude associated with a colder climate. The Gonghe Basin sandy land is located in the Qinghai-Tibet Plateau, and its annual mean temperature (2.4°C) is lower than that of the Tengger Desert (7.8°C) and Gurbantunggut Desert (5–5.7°C). Thus, the high relative abundance of Bacteroidetes in our study could be explained by the relatively colder environmental conditions of the Gonghe Basin. All these environmental variables may underpin differences in BSC bacterial communities between the alpine sandy land and other regions.

### 4.2. The change of bacterial communities with BSC development

MBC and MBN significantly increased with BSC development (Supplementary Figure S1), which was similar to the findings of a study conducted in the Tengger Desert (Yang et al., 2017) and Gurbantunggut Desert (Zhang et al., 2015). These results could be attributed to two potential reasons. First, with the development of BSCs, the thickness of the crusts increased, and the influence of wind erosion on the microbial community gradually weakened. This provided a relatively stable environment for the survival and reproduction of microbes. Second, the organic carbon and water content exerted the strongest influence on microbial biomass, including MBC and MBN (Xu et al., 2013). As the results showed, both the total organic carbon and water content significantly increased with the development of BSCs (*p* < 0.05, Table 1), resulting in higher bacterial biomass in this study.

Through the comparison of diversity based on OTUs, we found that the abundance and diversity of the bacterial community significantly increased with the development of BSCs and peaked in the moss crusts (Figure 3). Although similar holistic surveys of microbial diversity in different developmental stages of BSCs have been conducted in a few studies, there is evidence that the bacterial diversity index significantly increased with BSC development. For example, Mogul et al. (2017) recorded the bacterial diversity of early algae crusts in different land cover regions and showed that it increased with the development of BSCs in the central Mojave Desert. Li and Zhang (2017) observed that the diversity of the bacterial community was higher in lichen crusts than in algae crusts. Evidence from previous study suggested that microbial diversity positively influences multiple ecosystem functions related to nutrient cycling, and greater microbial community diversity support multiple functions simultaneously (Cameron et al., 2019). Thus, with the development of BSCs, richer and more diverse bacterial communities in our study may provide more functional attributes.

The bacterial community composition significantly changed with BSC development (Figure 4). The relative abundance of eutrophic groups, such as Actinobacteria, Acidobacteria, Bacteroidetes, and Planctomycetes, significantly increased with the development of BSCs (Supplementary Table S2). The increase in the abundance of these phyla could be explained by the improvement in nutritional conditions. Notably, most Actinobacteria are mycelial and may play a major role in maintaining the structure of BSCs in the later developmental stages (Maier et al., 2016). Meanwhile, we suggested that the high abundance of Actinobacteria in lichen and moss crusts may imply that some members contribute to primary productivity. Based on other findings from the Antarctic desert surface soil communities, the primary producers were proposed to be Actinobacteria (Mukan et al., 2017). Additionally, findings by Větrovsky et al. (2014) indicated that, Actinobacteria members can degrade complex compounds, such as polysaccharides and phenolic compounds, and improve the nutritional status of BSCs. Acidobacteria are heterotrophic bacteria with highly diverse functions (Lauber et al., 2009). As acidophilic bacteria, Acidobacteria had the greatest relative abundance in moss crusts with the lowest pH value in our study (Table 1). Bacteroidetes can secrete several exopolysaccharides, and the increase in their relative abundance may be related to the increase in fine particulate matter and plant growth in the later stages of BSC development (Zhang et al., 2018). In contrast, highly stress-resistant oligotrophic groups (Barnard et al., 2013), such as Proteobacteria and Firmicutes, were abundant in bare sand and microbial crusts, and their relative abundances decreased with further development (Supplementary Table S2). Gundlapally and Garcia-Pichel (2006) recorded that bacteria from phylum Proteobacteria can secrete extracellular polysaccharides to bind sand grains. These Proteobacteria were also shown to be the major contributors to N-fixation under nitrogen restriction conditions for BSC formation (Gundlapally and Garcia-Pichel, 2006; Zhou et al., 2020). The dominance of Firmicutes in the early stages of BSCs can be explained by its endospore-forming ability and the high G + C content, which enables them to survive in challenging dry conditions in bare sand and early stages of BSC development (Schimel et al., 2007). In addition, both Proteobacteria and Firmicutes showed a negative correlation with the soil water content, total carbon, and total organic carbon in our study (Figure 6), indicating that these bacteria can survive in an environment with low nutrition and water deficiency. This explains why their relative abundances were found to be higher in bare sand and microbial crusts. As the important oxygen-producing photosynthetic organism in BSCs, Cyanobacteria play the role of primary producers (Zhang et al., 2016). We found that the relative abundance of Cyanobacteria peaked in the algae crusts and decreased in the moss crusts (Supplementary Table S2). This may be related to the fact that the density and thickness of the pseudo roots of bryophytes are higher in the moss crusts, which affect the living space of Cyanobacteria. Additionally, members of the Cyanobacteria genus *Microcoleus* were also more abundant in algae crusts than in other stages (Figure 4B and Supplementary Table S2), and previous studies have shown that it can bind soil particles using filament bundles (Weber et al., 2016).

### 4.3. Relationships between bacterial communities and environmental variables of BSCs

In general, microbial communities can respond more rapidly than plant communities to change conditions during succession, and they emit early signals of the recovery trajectory. Findings from our study showed that the bacterial community composition of BSCs was significantly affected by the SWC, pH, TC, TOC, TN, NO_3_^−^, TP, and soil texture, among which SWC exerted the strongest effect on bacterial composition (*p* < 0.05; Table 2). This finding was consistent with the results reported by Zhang et al. (2018). According to previous studies, in arid and semi-arid regions, water availability and soil nutrient content are the key limiting factors affecting microbial communities (Yahdjian et al., 2011; Cheng et al., 2022). In the early stage of BSC formation, the bacterial community was restricted by water and nutrients. As the water and nutrient conditions gradually improved with the development of BSCs, the composition of the bacterial community gradually changed. In addition, in late developmental stages of BSCs, the increase in water content makes nutrients (Table 1), such as nitrate nitrogen, more easily dissoluble and utilizable by bacteria. This has a significant impact on bacterial community composition.

We also suggest that the bacterial community composition is a sensitive indicator of the water and nutrient conditions of BSCs at different developmental stages. Changes in water and nutrient conditions during BSC development are the primary reason for differences in bacterial communities in different developmental stages of BSC. In addition, different bacterial groups exhibit different responses to environmental processes in BSCs (Figure 6). This phenomenon may be attributed to the unique ecological niche of each group. Findings from previous studies have suggested that niches play a crucial role in the construction of bacterial community structures (Dumbrell et al., 2010; Yao et al., 2014). Therefore, changes in the bacterial community composition with BSC development may be attributed to the fact that BSCs at different developmental stages select species that are better adapted to a particular ecological niche, thus outperforming less adapted species in competition.

## 5. Conclusion

In this study, we identified the changes in bacterial communities with the development of BSCs in the Gonghe basin. Our findings provide novel insights that can help understand the development mechanism and ecological function of BSCs in the alpine sandy areas. We found that Acidobacteria and Bacteroidetes were abundant in different developmental stages of BSCs in this region. With the development of BSCs, the diversity of the bacterial community significantly increased, which is essential for maintaining various ecological functions during BSC development. As the water and nutrient conditions improved with BSC development, the bacterial community composition significantly changed, with an increase in the abundance of copiotrophic taxa and a decrease in the abundance of oligotrophic taxa. As different ecological functions are performed by each group of microbes, our results indicate potential changes in the ecological functions of bacterial communities with BSC development. These results further suggest that the soil water and nutrient contents are key factors driving the shift in bacterial communities with BSC development. Given the predominant role of microbes in BSC formation and development, our findings improve our understanding of the BSC developmental mechanisms and ecological functions, helping us assess the impact of climate change on Alpine desert ecosystems. We propose that the BSC development status can be used as an indicator to evaluate the degradation or health of alpine sandy land.

## Data availability statement

Publicly available datasets were analyzed in this study. This data can be found here: Sequence reads generated in this study were archived in the sequence read archive database of the National Center for Biotechnology Information under accession number PRJNA902024.

## Author contributions

HZ designed the experiments. HZ and YL conducted the sample collection and sample site survey. HZ, YL, and LL performed the experiments and analyzed the data. HZ wrote the manuscript along with YL and LL. All authors reviewed the article.

## Funding

This work was supported by the Natural Science Foundation of Qinghai Province (2022-ZJ-959Q) and the Open Project of State Key Laboratory of Plateau Ecology and Agriculture, Qinghai University (2021-ZZ-04).

## Conflict of interest

The authors declare that the research was conducted in the absence of any commercial or financial relationships that could be construed as a potential conflict of interest.

## Publisher’s note

All claims expressed in this article are solely those of the authors and do not necessarily represent those of their affiliated organizations, or those of the publisher, the editors and the reviewers. Any product that may be evaluated in this article, or claim that may be made by its manufacturer, is not guaranteed or endorsed by the publisher.

## Footnotes

^1^ http://qiime.org/
