# Supplementary material for: Biological soil crust development affects bacterial communities in the Caragana microphylla community in alpine sandy areas
Source: Front Microbiol. 2023 Mar 15;14:1106739. doi: 10.3389/fmicb.2023.1106739 (PMC10050341; doi:10.3389/fmicb.2023.1106739)
Supplement: Supplementary file 1 [file Data_Sheet_1.docx]

Supplementary Material

**Biological soil crust development affects bacterial communities in the Caragana microphylla community in Alpine sandy areas**

**Hong Zhou, Lun Li,** **Yunxiang Liu***

*** Correspondence:** Yunxiang Liu: [17791394452@163.com](mailto:17791394452@163.com)

# Supplementary Figures and Tables

## Supplementary Figures


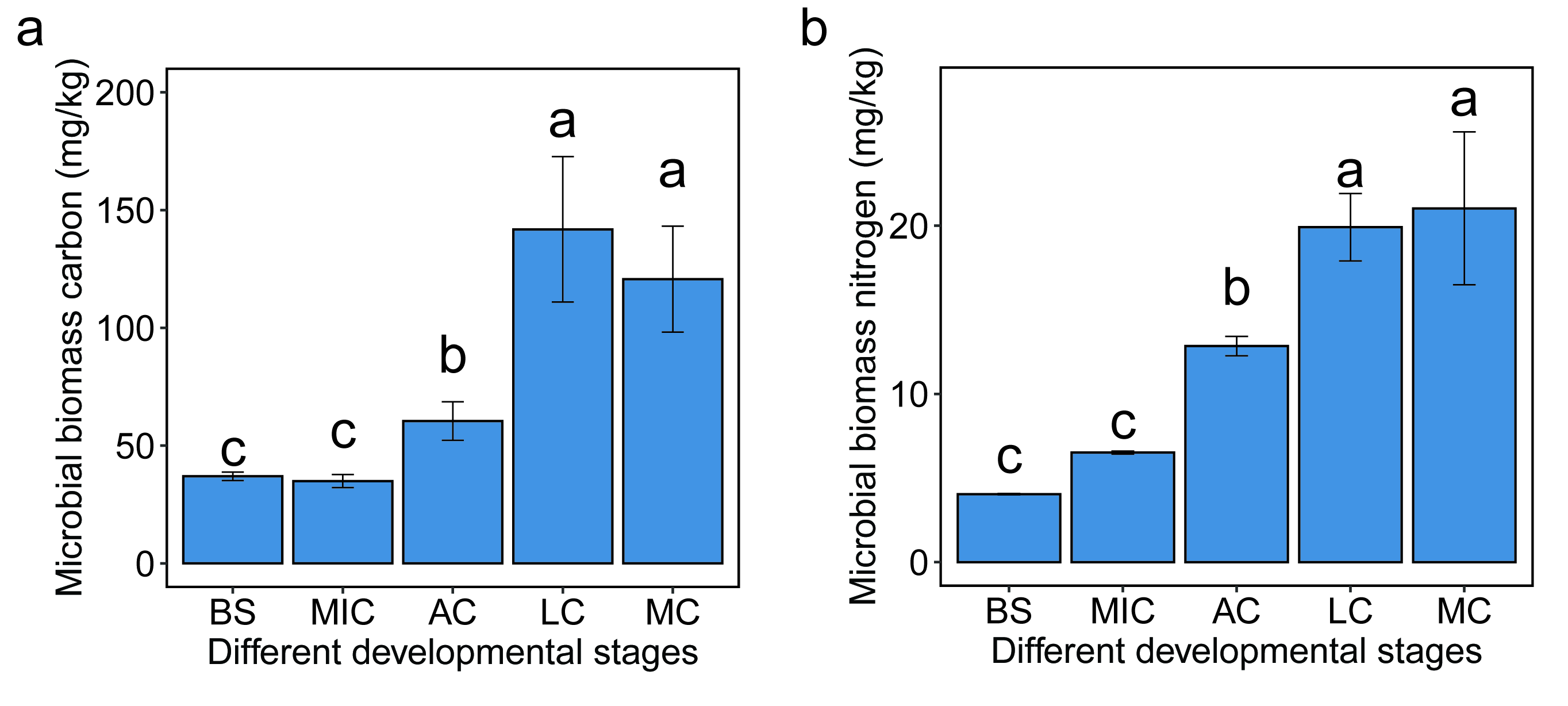


**Supplementary Figure S1.** Microbial biomass carbon (a) and microbial biomass nitrogen (b) contents in the different developmental stages of biological soil crusts. BS = bare sand; MIC = microbial crusts; AC = algae crusts; LC= lichen crusts; MC = moss crusts. Different letters indicate significant differences at P < 0.05. Error bars show SE (n = 8)

## Supplementary Tables

**Supplementary Table S1.** PerMANOVA (pairwise comparisons between treatments) to evaluate variations in the bacterial and fungal community structures. Significant P values are shown in bold.

| **Groups** | **R^2^** | ***P*** |
| --- | --- | --- |
| BS-MIC | 0.262 | **0.005** |
| BS-AC | 0.309 | **0.001** |
| BS-LC | 0.325 | **0.003** |
| BS-MC | 0.461 | **0.001** |
| MIC-AC | 0.274 | **0.011** |
| MIC-LC | 0.514 | **0.001** |
| MIC-MC | 0.323 | **0.001** |
| AC-LC | 0.168 | **0.014** |
| AC-MC | 0.221 | **0.002** |
| LC-MC | 0.521 | **0.017** |
| BS-MIC-AC-LC- MC | 0.422 | **0.001** |

BS = bare sand; MIC = microbial crusts; AC = algae crusts; LC = lichen crusts; MC = moss crusts.

**Supplementary Table S2.** Results of one-way ANOVA of the relative abundances of microbial phyla and genera (≥ 1%) in the different developmental stages of BSCs. The mean value and standard error (n = 3) are shown in the right columns of the table. P values reflecting statistical significance are shown in boldfaced font. Lowercase letters in the right column of the table indicate significant differences observed after water addition (P < 0.05).

| **level** | **Taxa groups** | **Bare sand** | **Microbial crusts** | **Algae crusts** | **Lichen crusts** | **Moss crusts** |
| --- | --- | --- | --- | --- | --- | --- |
| **Phylum** | *Actinobacteria* | 7.77±2.79**c** | 13.44±1.48**b** | 18.29±1.23**a** | 17.38±2.22**a** | 18.55±3.60**a** |
|  | *Acidobacteria* | 6.27±1.05**c** | 6.51±1.05**c** | 8.36±0.35**b** | 11.29±2.45**b** | 20.80±1.75**a** |
|  | *Bacteroidetes* | 12.80±0.79**c** | 11.81±1.99**c** | 15.12±1.08**b** | 18.59±1.05**a** | 17.92±2.01**a** |
|  | *Verrucomicrobia* | 0.86±0.10**d** | 1.57±0.23**c** | 1.59±0.45**c** | 1.91±0.29**b** | 4.63±1.50**a** |
|  | *Gemmatimonadetes* | 1.52±0.24**c** | 1.98±0.38**c** | 3.25±1.28**b** | 6.21±0.15**a** | 4.78±0.72**a** |
|  | *Planctomycetes* | 1.58±0.40**c** | 1.94±1.39**c** | 2.91±0.39**b** | 3.14±1.38**b** | 5.03±1.02**a** |
|  | *Cyanobacteria* | 0.42±0.05**d** | 12.99±1.86**b** | 13.30±1.97**a** | 3.24±0.99**c** | 2.33±0.36**c** |
|  | *Proteobacteria* | 32.09±7.02**a** | 28.94±2.33**b** | 27.01±2.25**b** | 19.96±1.81**c** | 15.01±1.20**d** |
|  | *Firmicutes* | 8.02±0.68**a** | 7.55±0.64**a** | 3.32±0.78**b** | 4.64±0.90**b** | 3.16±0.80**b** |
|  | *Chloroflexi* | 2.68±0.88 | 3.23±0.48 | 4.86±1.61 | 4.48±1.35 | 4.29±1.68 |
| **Genus** | *Ralstonia* | 2.00±0.34**a** | 0.44±0.10b | 0.13±0.01b | 0.16±0.02c | 0.12±0.06c |
|  | *Sphingomonas* | 2.65±0.21**a** | 1.98±0.26b | 1.67±0.17b | 1.43±0.25c | 1.28±0.08c |
|  | *RB41* | 1.25±0.09**c** | 1.16±0.18c | 1.14±0.20c | 2.48±0.63b | 9.25±1.53a |
|  | *Microcoleus* | 0.05±0.01d | 10.44±1.48b | 15.16±1.49a | 1.24±0.45c | 0.67±0.25c |
|  | *Bryobacter* | 0.29±0.13d | 0.95±0.21c | 1.31±0.18b | 0.91±0.25b | 1.47±0.27a |
|  | *Pseudonocardia* | 1.62±0.21 | 1.93±0.30 | 0.52±0.42 | 0.76±0.56 | 0.74±0.32 |
|  | *Microvirga* | 3.79±0.14 | 4.44±0.57 | 3.60±1.35 | 1.65±2.34 | 1.97±2.4 |
